# Supplementary material for: CD70-Targeted Micelles Enhance HIF2α siRNA Delivery and Inhibit Oncogenic Functions in Patient-Derived Clear Cell Renal Carcinoma Cells
Source: Molecules. 2022 Dec 2;27(23):8457. doi: 10.3390/molecules27238457 (PMC9738223; doi:10.3390/molecules27238457)
Supplement: Supplementary file 1 [file molecules-27-08457-s001.zip › molecules-1982809-supplementary.pdf]

Supplemental Data

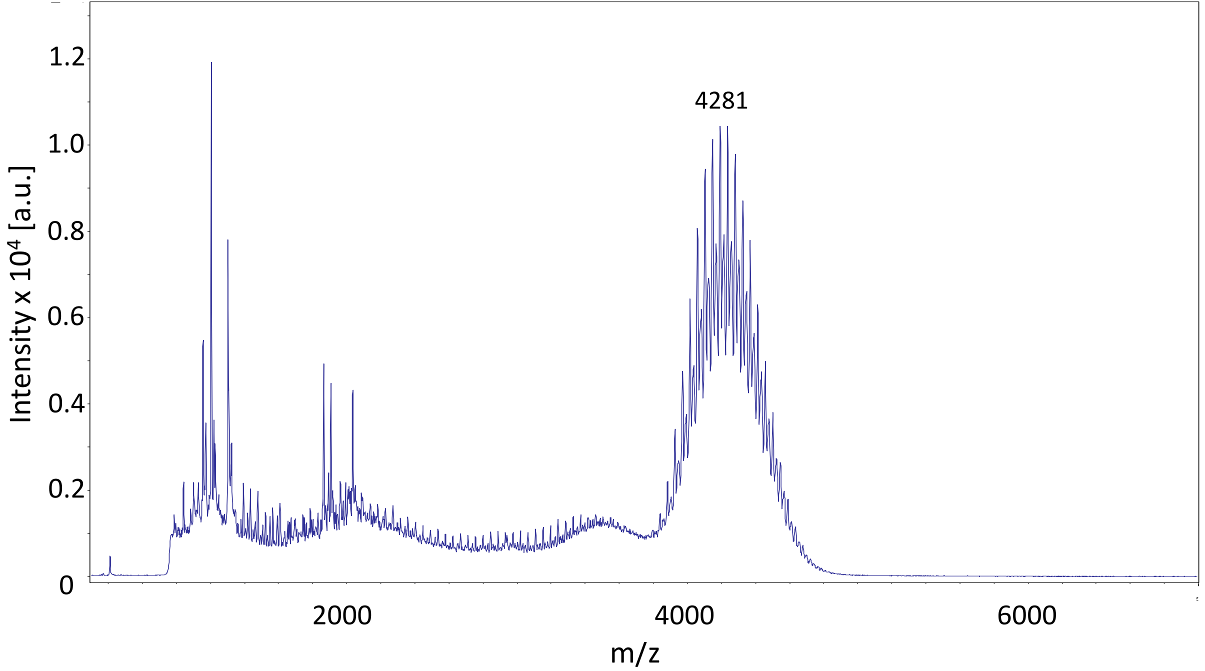

Figure S1. MALDI mass spectrum of CD27 PA. Expected m/z = 4343.

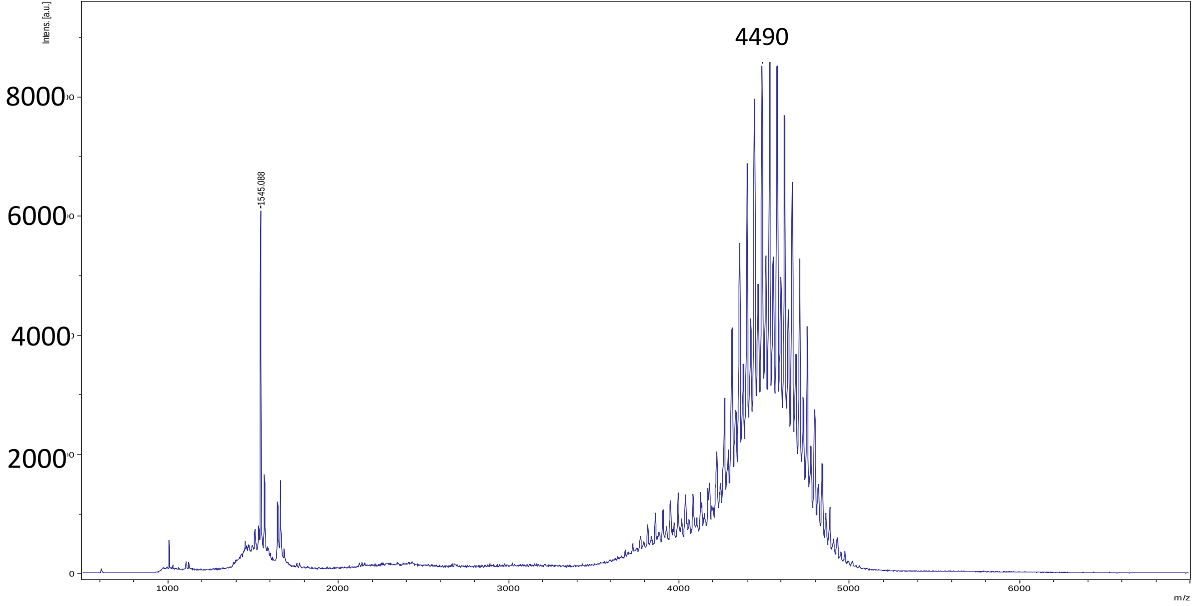

Figure S2. MALDI mass spectrum of scrCD27 PA. Expected m/z = 4343.

Table S1. Nanoparticle size

Sample	Size (nm)
HIF2α-CD27 PAM	13.8 ± 0.4
CD27 PAM	14.7 ± 0.5
HIF2α siRNA	N/A

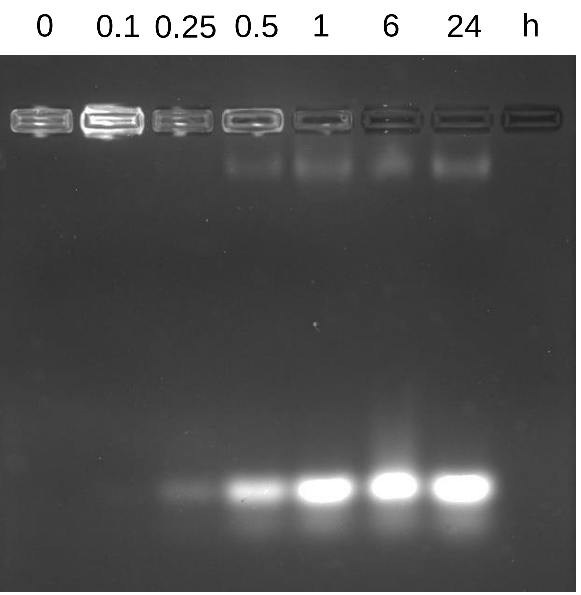

Figure S3. Agarose gel after siRNA release study.

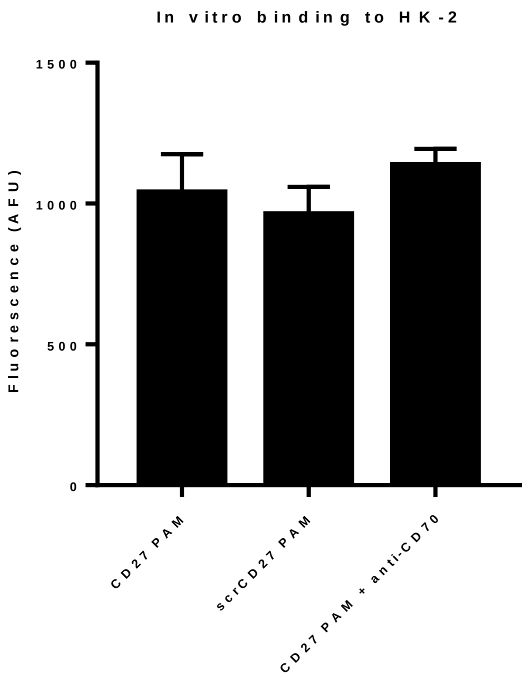

Figure S4. In vitro PAM binding to HK-2 cells.

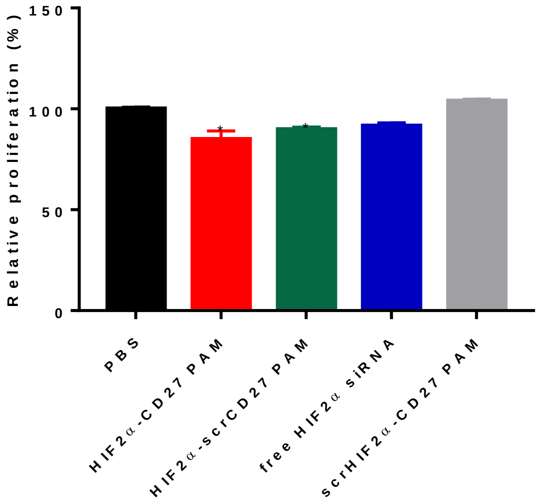

Figure S5. MTS assay of ccRCC cells 72h after transfection.

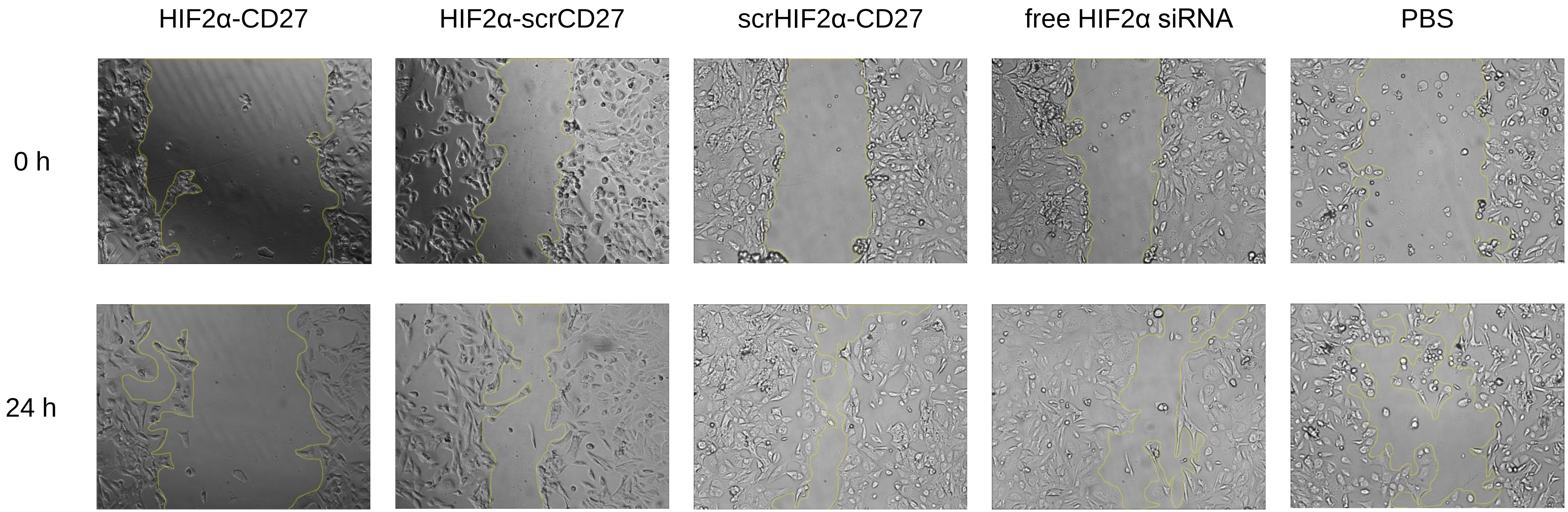

Figure S6. Representative image analysis slides for wound healing assay, timepoints 0h and 24h.

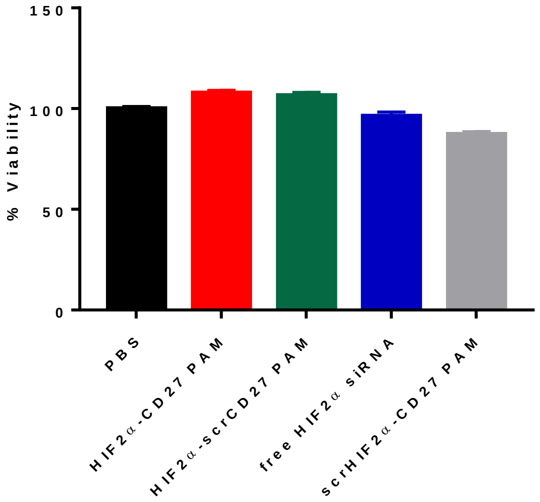

Figure S7. ccRCC viability following 48h transfection procedure.

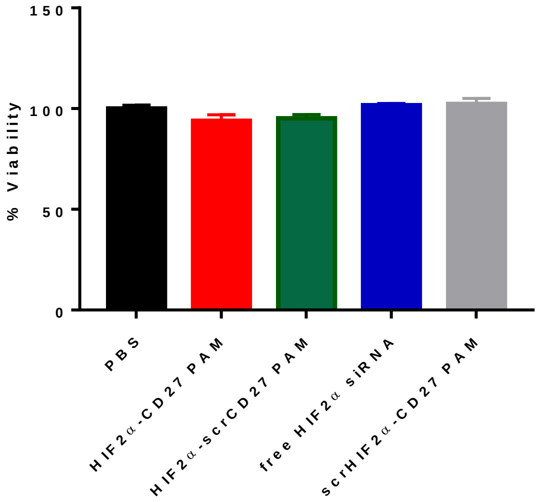

Figure S8. HK-2 cell viability following 48h transfection procedure.
